# Inhibition of Sphingosine Kinase-2 Suppresses Inflammation and Attenuates Graft Injury after Liver Transplantation in Rats

**DOI:** 10.1371/journal.pone.0041834

**Published:** 2012-07-25

**Authors:** Qinlong Liu, Hasibur Rehman, Yanjun Shi, Yasodha Krishnasamy, John J. Lemasters, Charles D. Smith, Zhi Zhong

**Affiliations:** 1 Departments of Pharmaceutical & Biomedical Sciences and Medical University of South Carolina, Charleston, South Carolina, United States of America; 2 Biochemistry & Molecular Biology, and Medical University of South Carolina, Charleston, South Carolina, United States of America; 3 Hollings Cancer Center, Medical University of South Carolina, Charleston, South Carolina, United States of America; 4 Apogee Biotechnology Corporation, Hummelstown, Pennsylvania, United States of America; The University of Hong Kong, Hong Kong

## Abstract

Inflammation mediates/promotes graft injury after liver transplantation (LT). This study investigated the roles of sphingosine kinase-2 (SK2) in inflammation after LT. Liver grafts were stored in UW solution with and without ABC294640 (100 µM), a selective inhibitor of SK2, before implantation. Hepatic sphingosine-1-phosphate (S1P) levels increased ∼4-fold after LT, which was blunted by 40% by ABC294640. Hepatic toll-like receptor-4 (TLR4) expression and nuclear factor-κB (NF-κB) p65 subunit phosphorylation elevated substantially after transplantation. The pro-inflammatory cytokines/chemokines tumor necrosis factor-α, interleukin-1β and C-X-C motif chemokine 10 mRNAs increased 5.9-fold, 6.1-fold and 16-fold, respectively following transplantation, while intrahepatic adhesion molecule-1 increased 5.7-fold and monocytes/macrophage and neutrophil infiltration and expansion of residential macrophage population increased 7.8–13.4 fold, indicating enhanced inflammation. CD4+ T cell infiltration and interferon-γ production also increased. ABC294640 blunted TLR4 expression by 60%, NF-κB activation by 84%, proinflammatory cytokine/chemokine production by 45–72%, adhesion molecule expression by 54% and infiltration of monocytes/macrophages and neutrophils by 62–67%. ABC294640 also largely blocked CD4+ T cell infiltration and interferon-γ production. Focal necrosis and apoptosis occurred after transplantation with serum alanine aminotransferase (ALT) reaching ∼6000 U/L and serum total bilirubin elevating to ∼1.5 mg/dL. Inhibition of SK2 by ABC294640 blunted necrosis by 57%, apoptosis by 74%, ALT release by ∼68%, and hyperbilirubinemia by 74%. Most importantly, ABC294640 also increased survival from ∼25% to ∼85%. In conclusion, SK2 plays an important role in hepatic inflammation responses and graft injury after cold storage/transplantation and represents a new therapeutic target for liver graft failure.

## Introduction

Despite significant progress in xenotransplantation, isolated hepatocyte transplantation, extracorporeal liver perfusion and liver assist devices, orthotopic liver transplantation (LT) remains the only proven therapy for end-stage liver diseases [1]–[5]. Initial poor function of liver grafts occurs in 10–50% of patients after LT [6]–[8]. Primary graft non-function (PNF) is a fatal complication after LT and requires retransplantation, which further exacerbates the already severe graft shortage [9], [10]. The mechanisms of PNF are not yet well understood, but ischemia/reperfusion (I/R) injury appears to play an essential role [8], [11]. Kupffer cell (KC) activation, free radical formation, toxic cytokine production, mitochondrial dysfunction and disturbed microcirculation lead to direct and indirect cytotoxicity and eventually graft failure [12]–[17].

The inflammation response is also of paramount importance in the development and progression of I/R injury [18], [19]. Multiple innate immune activation pathways exert pro- and anti-inflammatory functions. Diverse pathogen-associated and/or endogenous damage-associated molecular pattern (PAMP/DAMP) molecules generated during cell stress and I/R activate KC and dendritic cells [18], [19]. KCs produce cytotoxic free radicals and inflammatory cytokines after I/R and LT [19]–[21]. Increased proinflammatory cytokines, chemokines and other vasoactive/chemotactic mediators stimulate adhesion molecule expression, attract and activate leukocytes, and lead to microcirculation disturbances [12], [22], [23]. Activation of toll-like receptors (TLR), CD-14, MyD88, and nuclear factor-kappa B (NF-κB) pathways mediate proinflammatory responses [18], [24]–[27]. Complement also activates KCs and directly damages cell membranes during I/R [28], [29]. Recent studies showed that cross-talk between the innate immunity and adaptive immunity affects the progression of I/R injury [18], [24]. The inflammatory cytokine TNFα promotes migration of CD4+ T lymphocytes to the liver after I/R and CD4+ T cells, but not CD8+ T cells, appear to play a role in tissue damage by I/R [30], [31]. By contrast, IL-4, IL-10 and IL-13 were shown to alleviate I/R injury [32], [33].

In recent years, sphingolipid metabolism has emerged as a potential new therapeutic target for many diseases. Sphingosine kinases (SK1 and SK2) phosphorylate sphingosine, producing spingosine-1-phosphate (S1P) [34]–[36] which regulates a variety of important cell processes [36]–[38]. SK activation results in proinflammatory processes, including activation of inflammatory cells and increased expression of TNFα, NF-κB, cyclooxygenase-2, nitric oxide synthase (NOS) and adhesion molecules [36], [39]–[42].

Sphingolipid metabolism changes significantly in hypoxia and reperfusion. SK expression increases in cultured cells exposed to hypoxia, including hepatocytes [43]–[45]. S1P levels increase during myocardial infarction and after hepatic warm I/R [45], [46]. However, the role of SK in I/R injury remains controversial. Deficiency of the S1P receptor S1P3, decreases renal and pulmonary injury following I/R [47], [48], whereas adenoviral gene transfer of SK1 and treatment with S1P protect the heart against I/R injury [49], [50]. The effects of SKs in I/R injury may be organ specific, relating to the subtypes of SK or S1P receptors present in those tissues. Our recent work showed that ABC294640, a selective SK2 inhibitor, attenuates liver injury after warm I/R [45]. However, the role of SK2 in PNF remains unclear. Because SK activation can result in toxic cytokine production and inflammation, we tested the effects of SK2 inhibition by ABC294640 on inflammatory processes after LT in rats.

## Materials and Methods

### Liver Transplantation

Inbred male Lewis rats (200–250 g) were used in LT experiments to exclude immunological interference. LT was performed under isofluorane anesthesia using the rearterialized two-cuff technique described elsewhere [18]. After explantation, venous cuffs prepared from 14-gauge i.v. catheters were placed over the subhepatic vena cava and the portal vein, and each graft was stored in University of Wisconsin (UW) storage solution (Bridge to Life, Ltd., Columbia, SC) at 0–1°C for 18 hours. ABC294640 (see structure in Ref. [45], Apogee Biotechnology Corporation, Hummelstown, PA), a specific SK2 inhibitor [51], is soluble in the UW solution up to 100 µM. In preliminary studies, we explored the effects of ABC294640 ranging from 20 µM to 100 µM and maximal protection was observed with 100 µM. Therefore, ABC294640 with a final concentration of 100 µM or an equal volume of vehicle (DMSO) was added to the cold storage solution in this study.

For implantation, after dividing the bile duct at the hilum and clamping the suprahepatic and subhepatic vena cava and portal vein, the liver of the recipient was removed. The donor organ was then rinsed with lactated Ringer’s solution, and implanted by connecting the suprahepatic vena cava with a 7–0 Prolene (Ethicon, Somerville, NJ) running suture. The portal vein and subhepatic inferior cava were connected by insertion of cuffs and secured using a 6–0 silk suture. Both the hepatic artery and the bile duct were connected using the intraluminal polyethylene splints (PE-10 and PE-50, respectively). Implantation surgery required less than 40 minutes, and portal vein clamping time was for 16 to 17 minutes. For sham operation, ligaments around the liver were dissected, and 40 minutes later, the abdominal wall was closed with running suture without transplantation. Rats were observed for 28 days after surgery for survival.

### Measurement of Serum Alanine Aminotransferase (ALT) and Total Bilirubin

Our previous experiments showed that inflammatory responses, clinical chemistry and cell death all become overt/remain elevated at ∼18 h after transplantation. Therefore, blood and liver samples were collected at 18 h. Under pentobarbital anesthesia (50 mg/kg, ip), blood samples were collected from the inferior vena cava. Serum was obtained by centrifugation and stored at −80°C. Serum ALT and total bilirubin were determined using analytical kits from Pointe Scientific (Lincoln Park, MI).

### Histology and Immunohistochemistry

Liver grafts were collected 18 h after implantation under pentobarbital anesthesia. For each rat, the liver was rinsed with 10 mL normal saline and perfusion-fixed with 10 mL 4% paraformaldehyde (pH. 7.0; VWR Inc., West Chester, PA) through a 24-gauge i.v. catheter inserted into to the portal vein. Tissue blocks were imbedded in paraffin after immersion in 4% paraformaldehyde for 48 hours. After hematoxylin-eosin (H&E) staining, liver sections were analyzed microscopically for pathology. Ten random images were captured in a blind manner from each slide using a microscope (Zeiss Axiovert 100 microscope, Thornwood, NY) and a 10x objective lens. Necrotic areas were quantified by image analysis using an IPlab 3.7v software (BD Biosciences, Rockville, MD) as described elsewhere [17].

Apoptosis was assessed by terminal deoxynucleotidyl transferase dUTP nick-end labeling (TUNEL) using an in situ cell death detection kit from Roche Diagnostics (Penzberg, Germany) [52]. TUNEL-positive and negative cells were counted in a blinded manner in 10 randomly selected fields per slide under a Nikon Optiphot-2 microscope (Nikon Instruments Inc., Melville, NY) using a 40× objective lens.

ED1, a marker of infiltrating monocyte/macrophages, was detected by immunohistochemical staining. Liver sections were deparaffinized and rehydrated as described elsewhere [52]. Hydrated sections were then incubated with mouse anti-ED1 monoclonal antibodies (Serotek, Raleigh, NC) at a 1∶500 dilution in 0.1 M phosphate buffer-Tween for 30 min at room temperature followed by exposure to peroxidase-conjugated anti-mouse IgG_1_ antibody (DAKO Corp., Carpinteria, CA) for 15 min at room temperature, and 3,3′-diaminobenzidine chromagen was added as the peroxidase substrate. After the immunostaining procedure, a light counterstain of Meyer’s hematoxylin was applied so ED1-expressing cells could be identified easily. ED1-positive cells were counted in a blinded manner in 10 randomly selected fields per slide using a 40× objective.

### Detection of Sphingosine-1-Phosphate

SK activity was assessed by S1P production. Livers were homogenized in ice-cold 50 mM Tris buffer (pH 7.4) containing 0.25 M sucrose, 25 mM KCl, 0.5 mM EDTA, and 1% phosphatase inhibitor cocktail (Sigma-Aldrich, St. Louis, MO). After centrifugation of liver homogenates at 2,500×g for 10 minutes at 4°C, S1P in supernatants was determined using an enzyme-linked immunosorbent assay kit from Echelon Inc. (Salt Lake City, UT). Protein was determined using a Protein Assay Kit from Bio-Rad Laboratory (Hercules, CA).

### Immunoblotting

Livers were harvested at 18 h after transplantation or sham operation, and immunoblotting was performed as described elsewhere [17] using specific antibodies against cleaved caspase-3 (Cell Signaling Technology, Danvers, MA), toll-like receptor-4 (TLR4, Abcam, Cambridge, MA), NF-κB p65, phosphorylated NF-κB p65 (Ser 536), ED2 (Santa Cruz Biotechnology, Santa Cruz, CA), intracellular adhesion molecule-1 (ICAM-1; BD Biosciences Pharmingen, San Diego, CA), myeloperoxidase (MPO; DAKO Corp., Carpinteria, CA), CD4 (Origene Technologies, Rockville, MD), interferon-γ (IFNγ, Biolegend, San Diego, CA) at concentrations of 1∶500–1∶1000**,** or actin (Cell Signaling Technology; 1∶3000) at 4°C over night.

### Detection of TNFα, IL-1β and CXCL-10 mRNAs by Quantitative Real-Time PCR

The mRNAs of tumor necrosis factor-α (TNFα), interleukin-1β (IL-1β) and C-X-C motif chemokine 10 (CXCL-10) were detected by quantitative real-time PCR, as described elsewhere [52], using the primers listed in Table 1. The abundance of mRNAs was normalized against hypoxanthine phospho-ribosyl-transferase (HPRT) using the ΔΔ*Ct* method.

**Table 1:** Real-Time PCR Primers.

mRNAs		Primers
**IL-1β**	Forward:	5′-AGCAGCTTTCGACAGTGAGGAGAA -3′
	Reverse:	5′-TCTCCACAGCCACAATGAGTGTGACA-3′
**TNF-α**	Forward:	5′-CAGACCCTCACACTCAGATCATCTT-3′
	Reverse:	5′-CAGAGCAATGACTCCAAAGTAGACCT-3′
**CXCL-10**	Forward:	5′- TGCAAGTCTATCCTGTCCGCATGT-3′
	Reverse:	5′- TAGACCTTCTTTGGCTCACCGCTT-3′
**HPRT**	Forward:	5′- TCGAAGTGTTGGATACAGGCCAGA-3′
	Reverse:	5′-TACTGGCCACATCAACAGGACTCT-3′

IL-1β, interleukin-1β; TNF-α, tumor necrosis factor; CXCL-10, C-X-C motif chemokine 10; HPRT, hypoxanthine phospho-ribosyl-transferase.

### Statistical Analysis

Groups were compared using ANOVA plus Student-Newman-Keul’s post-hoc test or Kaplan-Meier test using p<0.05 as the criterion of significance. Values are means ± SEM. There were 8 transplantations per group for survival experiments and 4 transplantations per group for all other parameters.

### Ethics Statement

All animals were given humane care in compliance with institutional guidelines using protocols approved by the Institutional Animal Care and Use Committee of the Medical University of South Carolina (AR#2841).

## Results

### Transplantation Increases S1P Production in the Liver: Prevention by ABC294640

**Figure 1:**
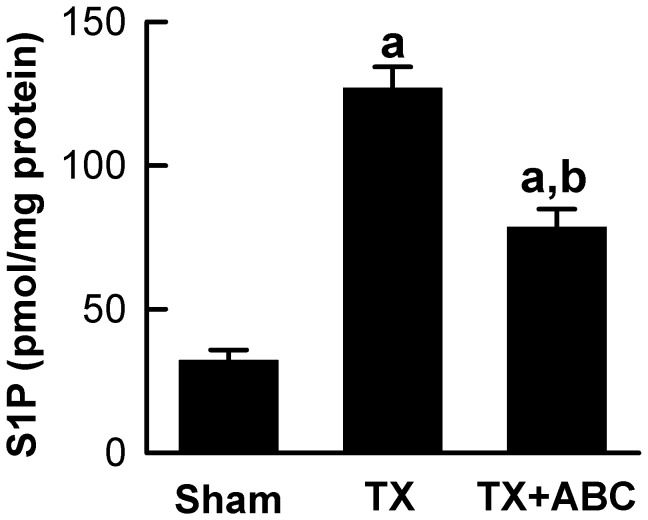
ABC294640 decreases S1P production after liver transplantation. Livers were harvested at 18 h after transplantation (TX) or sham-operation (Sham). Sphingosine-1-phosphate (S1P) in liver tissues was quantified by ELISA. ABC, ABC294640; a, p<0.05 vs sham; b, p<0.05 vs the TX group (n = 4 per group).

Our previous study showed that SK2 expression and activity increase after hepatic warm I/R [45]. Therefore, we investigated whether SK activity also increases after cold storage/transplantation. Hepatic S1P, the product of SK catalyzed reaction, increased from the basal levels of ∼30 pmol/mg protein to ∼127 pmol/mg protein at 18 h after transplantation (Fig. 1), indicating increased SK activity. ABC294640, a specific SK2 inhibitor that does not affect SK1 and other kinases [53], significantly decreased S1P formation after transplantation (Fig. 1).

### ABC294640 Prevents Toll-Like Receptor-4 Upregulation and NF-κB Activation after Liver Transplantation

**Figure 2:**
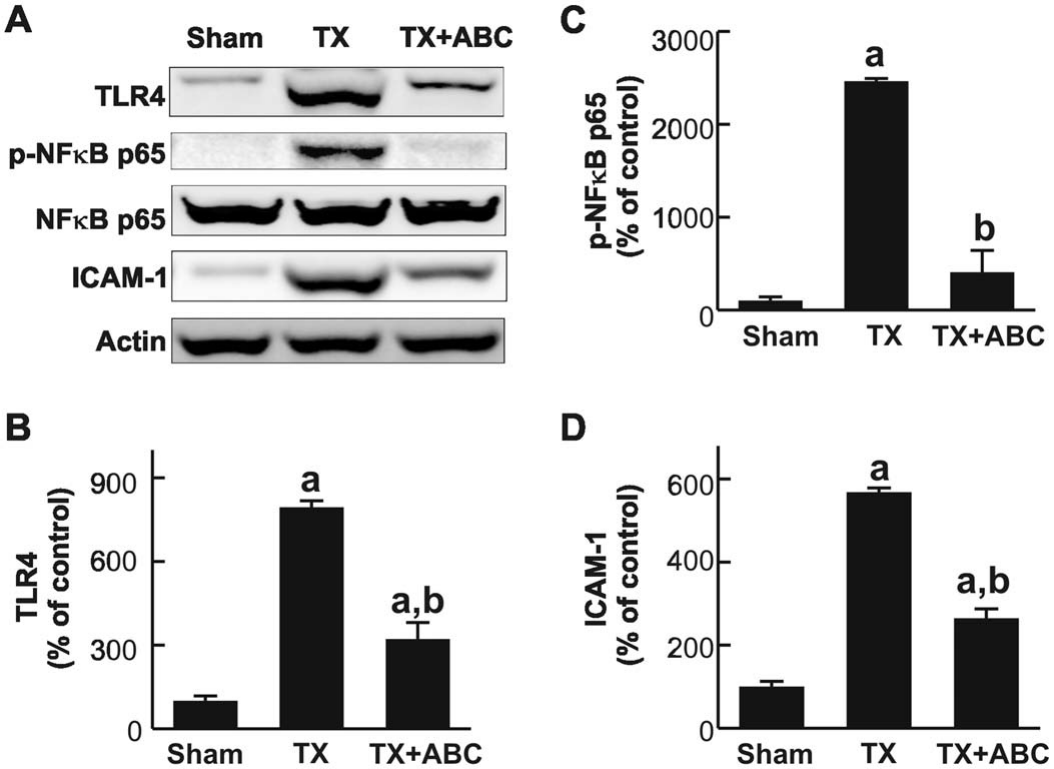
ABC294640 blunts TLR4 and ICAM-1 upregulation and NF-κB activation after liver transplantation. Livers were harvested at 18 h after transplantation (TX) or sham-operation (Sham). Levels of TLR4, NF-κB p65 subunit, phosphorylated NF-κB p65 subunit (p-NF-κB p65), intracellular adhesion molecule-1 (ICAM-1) and actin were determined by immunoblotting. Representative blot images are shown in **A**. Images were quantified by densitometry (**B–D**). ABC, ABC294640; a, p<0.05 vs sham; b, p<0.05 vs the TX group (n = 4 per group).

TLRs play a fundamental role in pathogen recognition and activation of innate immunity, leading to inflammatory responses. Therefore, we examined the effects of ABC294640 on expression of TLR4 which could be induced by increased lipopolysaccharide (LPS) in the blood or ROS after LT. TLR4 was expressed in low levels in the livers of sham-operated rats but increased ∼8-fold at 18 h after LT. Upregulation of TLR4 was blunted 60% by ABC294640 (Fig. 2A and 2B).

TLR4 confers LPS responsiveness by activation of NF-κB [27]. Total NF-κB p65 subunits remained unchanged after transplantation. By contrast, the phosphorylated p65 subunits of NF-κB were barely detectable in sham-operated rats but increased 25-fold 18 h after LT. ABC294640-treatement strongly blunted NF-κB activation by 84% (Fig. 2A and 2C).

### ABC294640 Prevents Adhesion Molecule Expression and Toxic Cytokine/Chemokine Formation after Liver Transplantation

ICAM-1 is a transmembrane glycoprotein that regulates neutrophil adhesion and transcellular migration [54]. When activated, leukocytes bind to endothelial cells via ICAM-1/LFA-1 interactions and then transmigrate into tissues. ICAM-1 was expressed at low levels in sham-operated livers but increased 5.7-fold at 18 h after transplantation. ABC294640 significantly decreased ICAM-1 expression by 54% (Fig. 2A and 2D).

**Figure 3:**
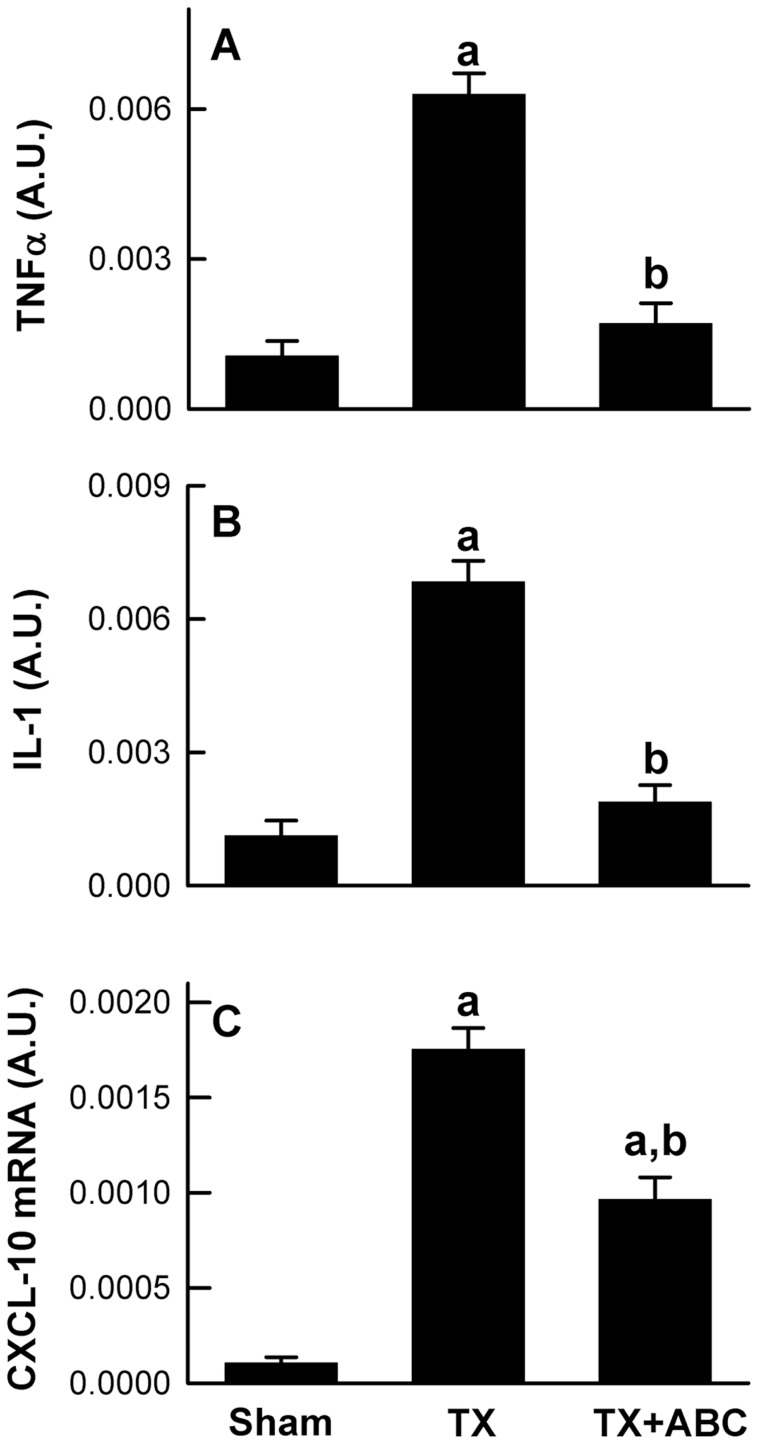
ABC294640 blunts TNFα, IL-1β and CXCL-10 mRNA increases after liver transplantation. Livers were harvested at 18 h after transplantation (TX) or sham-operation (Sham). Tumor necrosis factor-α (TNFα, **A**), interleukin-1β (IL-1β, **B**) and C-X-C motif chemokine 10 (CXCL-10, C) mRNAs in liver tissue were measured by quantitative real-time PCR. A.U., arbitrary units. ABC, ABC294640; a, p<0.05 vs sham; b, p<0.05 vs the TX group (n = 4 per group).

Toxic cytokines, e.g. TNFα, stimulate inflammatory processes and cause cell death. TNFα mRNA increased 5.9-fold after transplantation (Fig. 3A). The mRNA of IL-1β, another major inflammatory cytokine, increased 6.1-fold after transplantation (Fig. 3B). ABC294640-treatment inhibited the increases of TNFα and IL-1β mRNAs significantly (Fig. 3A and 3B).

C-X-C motif chemokine 10 (CXCL-10) belongs to the CXC chemokine family and plays important roles in chemoattraction of monocytes/macrophages, T cells, NK cells, and dendritic cells [55]. CXCL-10 mRNA increased ∼16-fold after transplantation, and ABC294640-treatment significantly blunted this increase in CXCL-10 mRNA by 45% (Fig. 3C).

### ABC294640 Decreases Leukocyte Recruitment and Reactive Oxygen Species Formation after Liver Transplantation

**Figure 4:**
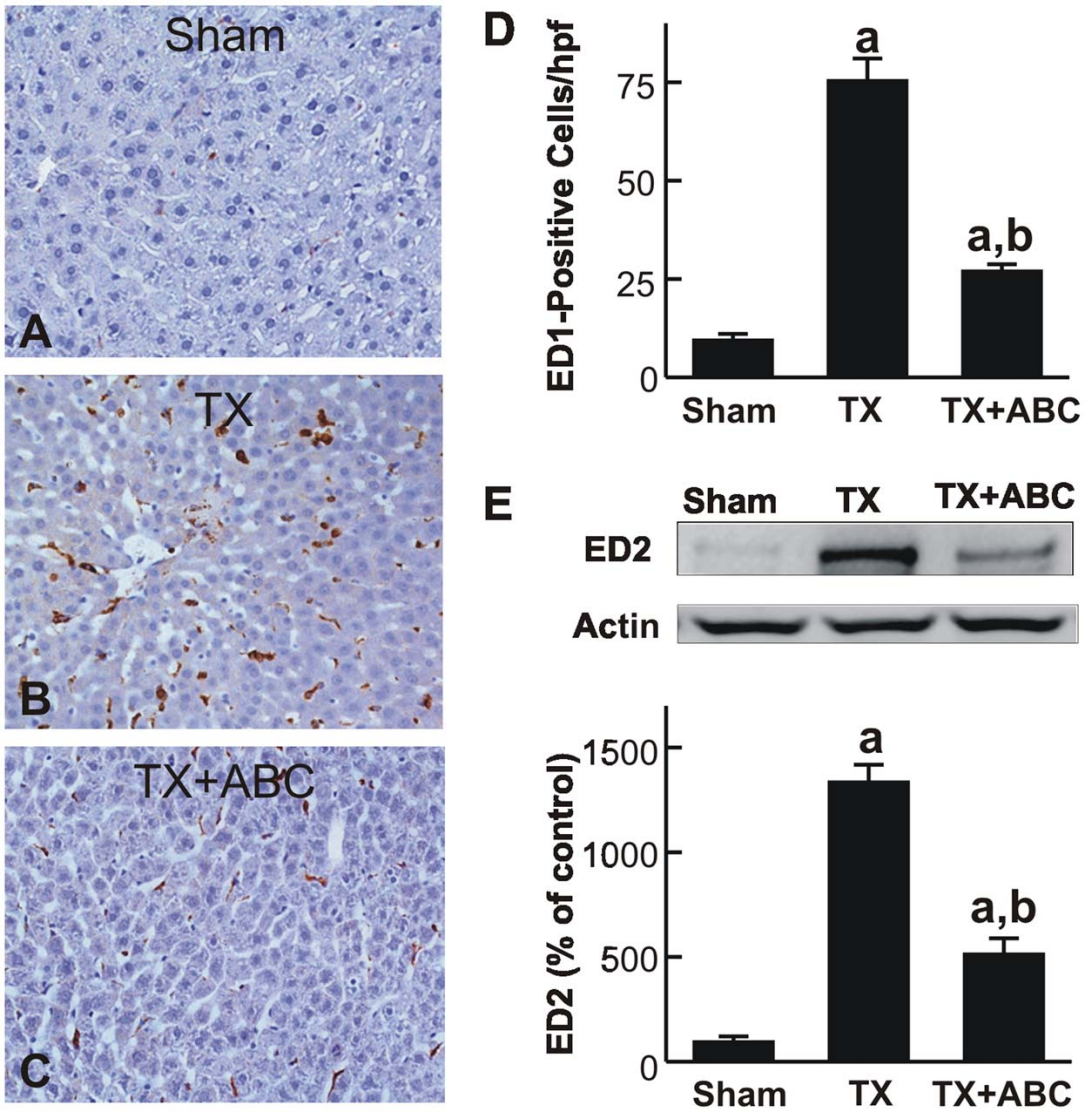
ABC294640 decreases monocyte/macrophage infiltration after liver transplantation. Livers were harvested at 18 h after transplantation (TX) or sham-operation (Sham). ED1 was detected immunohistochemically (**A–C**). ED1-positive cells were counted in a blinded manner in 10 randomly selected fields using a 40x objective lens (**D**). ED2 and actin in liver tissue were detected by immunoblotting. Representative blots and quantification by densitometry are shown in **E**. ABC, ABC294640; a, p<0.05 vs sham; b, p<0.05 vs the TX group (n = 4 per group).

ED1-positive cells and ED2 expression were measured to evaluate monocyte/macrophage infiltration and resident macrophage (KC) population expansion, respectively [56], [57]. ED1-positive cells increased from the basal level of ∼9 cells/hpf to ∼76 cells/hpf after transplantation (Fig. 4A, 4B and 4D). ED1-positive cells increased not only in the necrotic areas but also increased substantially in areas without visible cell death. However, ED1-positive cells were significantly reduced to only 27 cells/hpf in ABC294640-treated liver grafts (Fig. 4C and 4D). ED2 expression increased ∼13-fold after transplantation, indicating expansion of the resident macrophage population, and ABC294640-treatment significantly blunted the ED2 increases by ∼62% (Fig. 4E).

**Figure 5:**
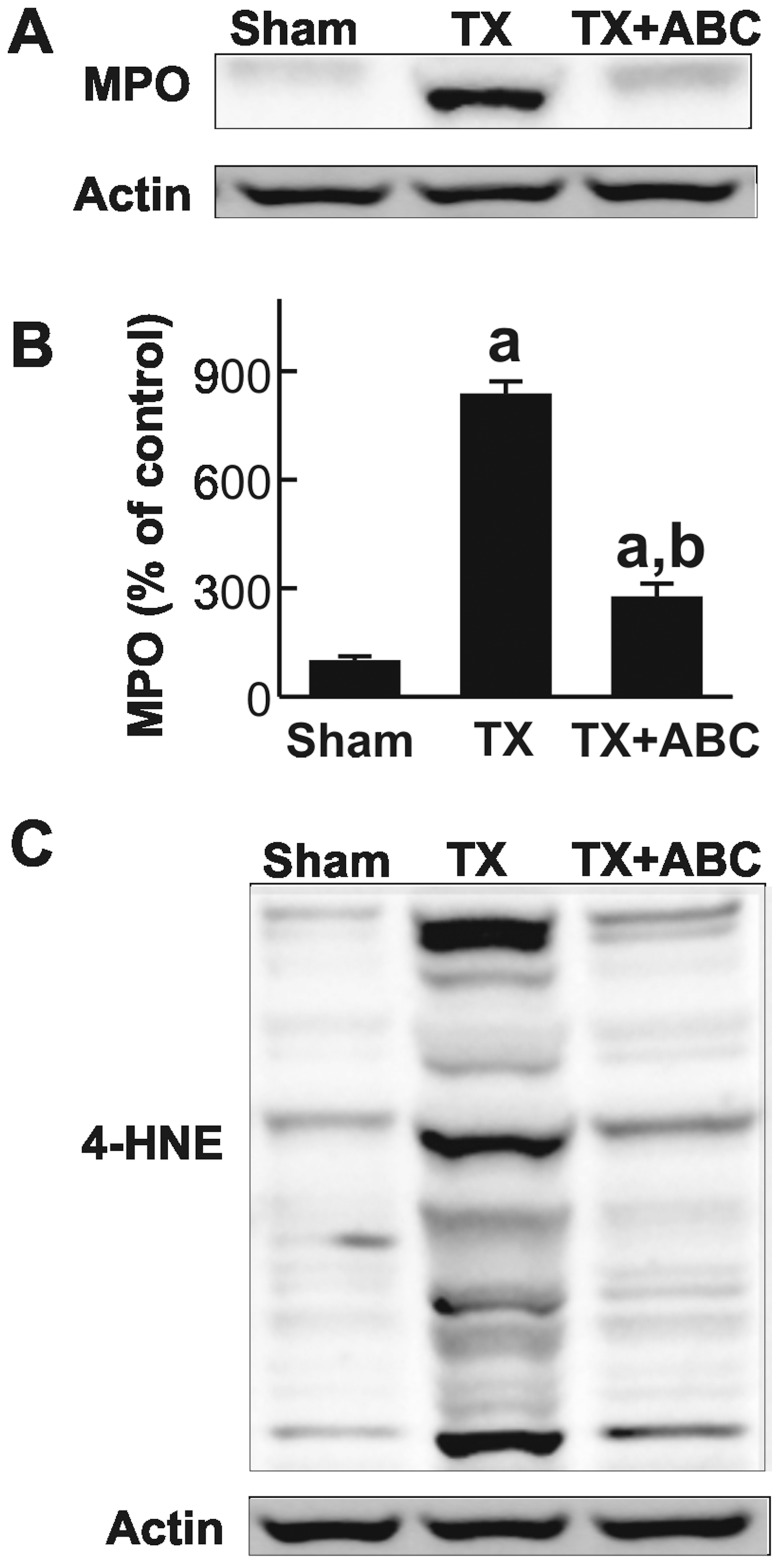
ABC294640 blunts increases in MPO and 4-HNE after liver transplantation. Livers were harvested at 18 h after transplantation (TX) or sham-operation (Sham). Myeloperoxidase (MPO, **A**), 4-hydroxynonenal adducts (4-HNE, C) and actin were determined by Western blotting. MPO images were quantified by densitometry (**B**). ABC, ABC294640; a, p<0.05 vs sham; b, p<0.05 vs the TX group (n = 4 per group).

MPO expression in the liver increased by 8.4-fold after transplantation, indicating infiltration of polymorphonuclear neutrophils (PMNs). ABC294640 significantly blunted increases in hepatic MPO levels after transplantation by 67% (Fig. 5A and 5B).

Both PMNs and KCs produce ROS, leading to tissue damage. Accordingly, we assessed 4-HNE expression, a marker of lipid peroxidation after transplantation, by western blotting (Fig. 5C). In the livers of sham-operated rats, only very weak 4-HNE-positive bands were detected. After LT, multiple strong 4-HNE-positive bands were detected, indicating formation of 4-HNE protein adducts. ABC294640 significantly blunted these increases of 4-HNE adducts. Thus, inhibition of SK2 reduced recruitment/expansion of a number of inflammatory cell populations and oxidative stress in the liver grafts after transplantation.

**Figure 6:**
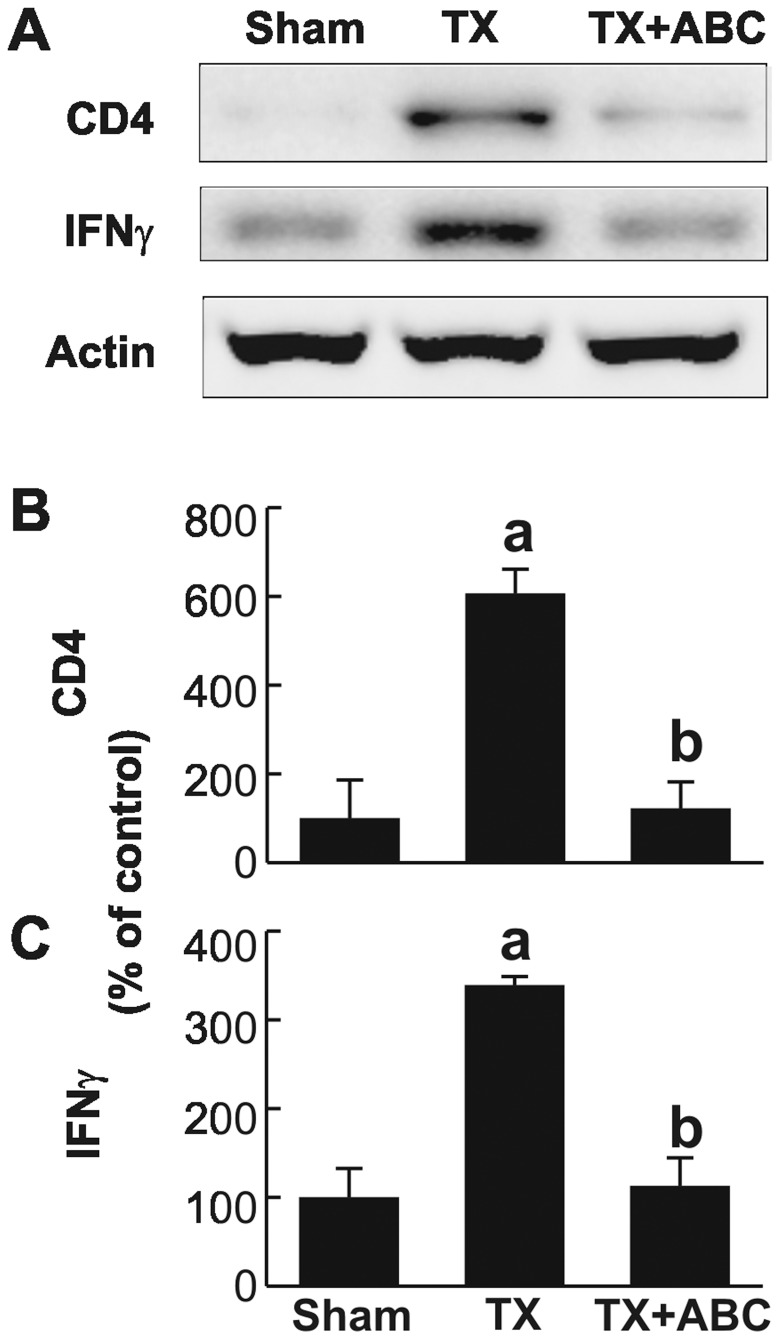
ABC294640 blunts CD4+ T Cell Infiltration and IFNγ Production after liver transplantation. Livers were harvested at 18 h after transplantation (TX) or sham-operation (Sham). CD4, IFNγ and actin were determined by immunoblotting and representative images are shown in **A**. CD4 and IFNγ images were quantified by densitometry (**B and C**). ABC, ABC294640; a, p<0.05 vs sham; b, p<0.05 vs the TX group (n = 4 per group).

Recent studies show that not only innate immunity but also adaptive immunity affects the progression of I/R injury, and CD4+ T cells appear to play a role in tissue damage by I/R [18], [24]. Thus, we investigated CD4+ T cell levels after transplantation. CD4 expression was barely detectable before transplantation but increased ∼6-fold after transplantation (Fig. 6A and 6B). This increase of CD4 was significantly reduced by ABC294640. IFNγ is produced by CD4+ T cells and NK cells [58] [18]. IFNγ was expressed at low levels in sham-operated rats but increased ∼3-fold after transplantation (Fig. 6A and 6C). ABC294640 also significantly decreased IFNγ expression. Therefore, ABC294640 also inhibits T cell recruitment and activation after transplantation.

### ABC294640 Attenuated Injury and Improved Graft Function after Transplantation

**Figure 7:**
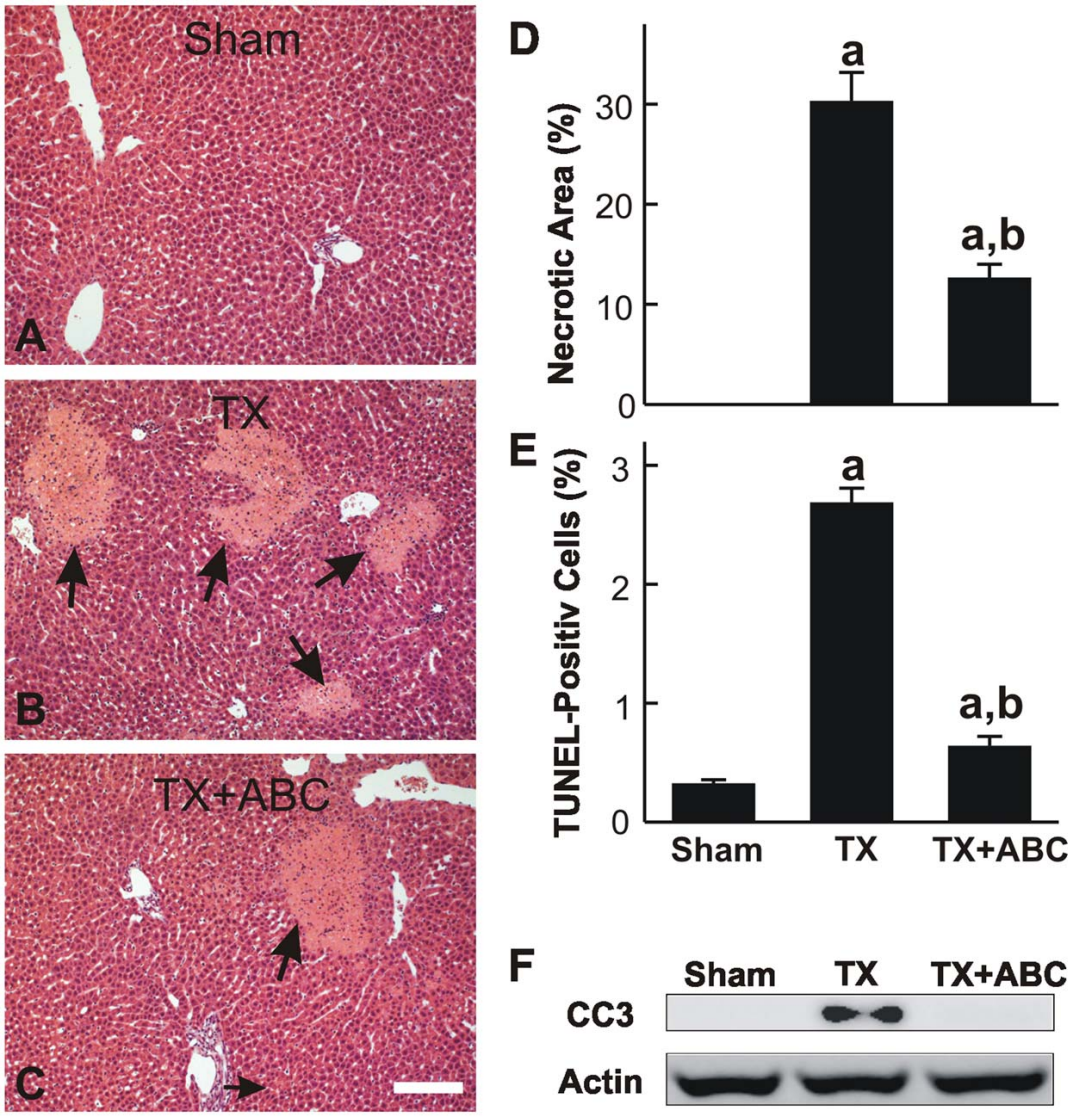
ABC294640 attenuates necrosis and apoptosis after liver transplantation. Livers were harvested at 18 h after transplantation (TX) or sham-operation (Sham). Liver slices were stained with H&E for assessment of necrosis. Representative images are shown in **A to C**. Arrows identify the necrotic areas. The bar is 100 µm. Necrotic areas were quantified by image analysis of 10 randomly selected fields (**D**). Apoptosis was assessed by TUNEL staining, and TUNEL-positive cells were counted in 10 randomly selected fields (**E**). Cleaved caspase-3 and actin were detected by immunoblotting (**F**). ABC, ABC294640; a, p<0.05 vs sham; b, p<0.05 vs the TX group (n = 4 per group).

Increased SK2 expression and S1P production were associated with liver injury after warm I/R [45]. Therefore, we assessed the effects of ABC294640 on liver injury and function after LT. After sham operation, no hepatic histopathological changes were observed (Fig. 7A). At 18 h after transplantation, focal necrosis was present in ∼30% of the liver tissue (Fig. 7B and 7D), and this was primarily localized in midzonal and pericentral regions (Fig. 7B). Pretreatment with ABC294640 attenuated transplantation-induced liver necrosis to ∼13% (Fig. 7C and 7D).

Apoptosis was evaluated by TUNEL staining (Fig. 7E). TUNEL-positive hepatocytes increased from a basal level of 0.3% to 2.7% at 18 h after transplantation. This increase in apoptosis was also significantly attenuated by ABC294640. Cleaved caspase-3 was barely detectable after sham-operation but increased after LT, confirming the occurrence of apoptosis (Fig. 7F). ABC294640-treatment blunted the activation of caspase-3. Together, these data demonstrate that cold storage/transplantation causes cell death in liver grafts, with necrosis being the predominant form of cell death over apoptosis. Furthermore, SK2 and S1P appear to play important roles in transplantation-induced liver injury.

### ABC294640 Improves Liver Function and Survival after Transplantation

**Figure 8:**
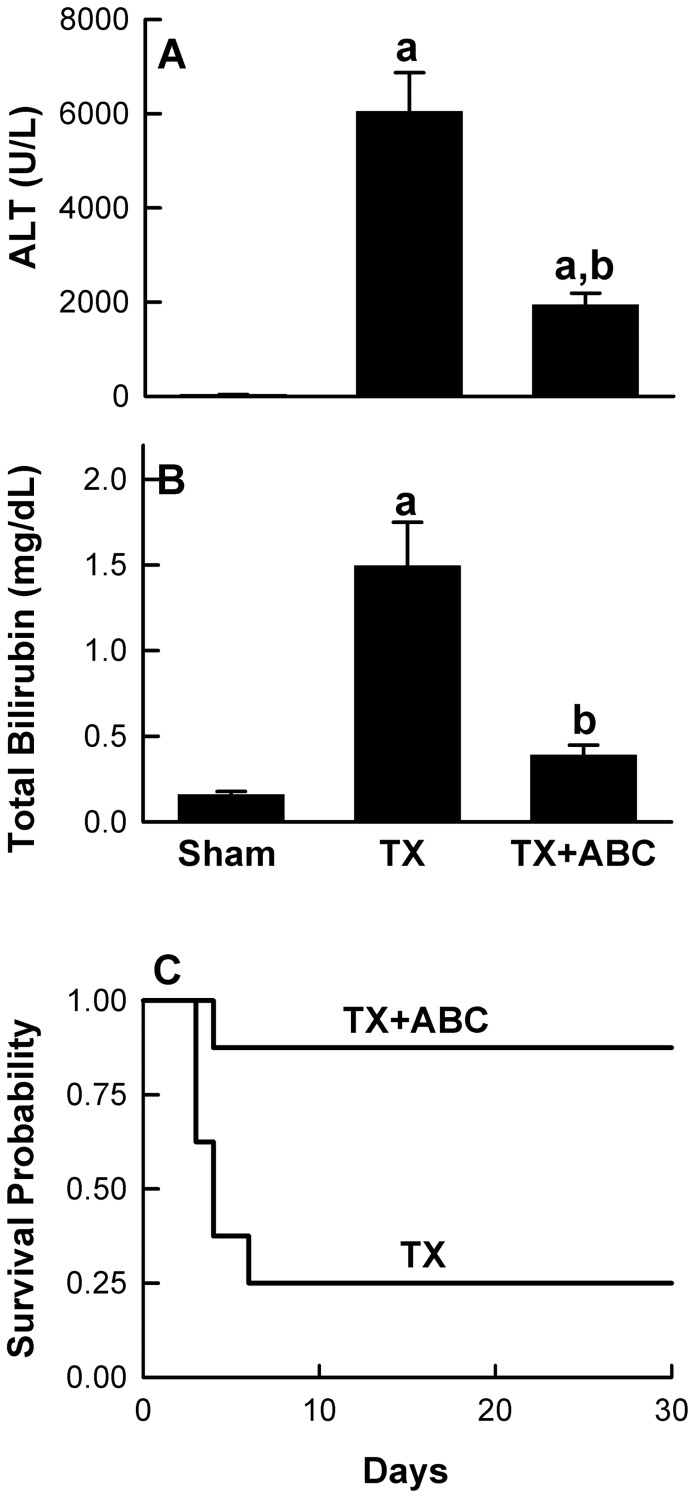
ABC294640 protects liver function and improves survival after liver transplantation. Blood samples were collected at 18 h after transplantation (TX) or sham-operation (Sham) for ALT (**A**) and total bilirubin measurement (**B**). ABC294640; a, p<0.05 vs sham; b, p<0.05 vs the TX group (n = 4 per group). Survival probabilities (**C**) after transplantation were significantly different between vehicle- and ABC294640-treated rats (n = 8 per group) by the Kaplan-Meier test.

After sham operation, serum ALT levels were 36 U/L, which increased to 6042 U/L 18 h after transplantation, indicating severe liver injury (Fig. 8A). ABC294640 significantly suppressed the ALT levels to ∼2,000 U/L, (Fig. 8A).

Serum bilirubin was ∼0.15 mg/dL before transplantation and increased ∼10-fold at 18 h after transplantation, confirming poor graft function (Fig. 8B). ABC294640-treatment significantly blunted hyperbilirubinemia after transplantation by 74% (Fig. 8B).

All rats survived after sham operation (data not shown). However, only 25% of the control rats survived for 28 days after transplantation, with death occurring mainly in the first 3 days (Fig. 8C). In marked contrast, the survival significantly increased to ∼85% in rats receiving ABC294640-pretreated grafts (Fig. 8C), indicating that ABC294640 markedly reduces the liver graft failure rate.

## Discussion

### SK2 Plays an Important Role in Liver Graft Injury after Transplantation

Despite important progression in stem cell studies and bioartificial organs in recent years, LT remains the treatment of choice for end-stage liver diseases [1]–[5], and PNF continues to be an important therapeutic target for improvement of LT outcome. I/R injury plays an essential role in PNF, but its mechanisms are not fully understood.

Sphingolipid metabolism produces important second messengers that regulate cell death, proliferation, and inflammation [36]–[38]. Two subtypes of SK (SK1 and SK2) exist in mammals. Many cytokines rapidly elevate cellular SK activity [35]–[37] and warm hepatic I/R increases SK2 expression in cultured cells and in vivo [45]. In recent years, a series of selective SK inhibitors have been identified. These inhibitors do not interfere with the ATP binding site on enzymes; therefore, their biological effects are unlikely mediated by off-target inhibition of other lipid and protein kinases [51], [59]. Our recent studies showed that ABC294640, a highly selective SK2 inhibitor, markedly decreased mitochondrial damage, blocked inflammatory processes, decreased liver injury and prevented acute liver failure after warm hepatic I/R [45]. These results suggest that SK2 activation is essential for hepatic I/R injury. Therefore, in this study we further explored the possibility of suppression of inflammation and attenuation of graft injury by SK2 inhibition after LT where cold I/R predominates. Indeed, ABC294640 treatment decreased liver graft injury (necrosis, apoptosis and ALT release), improved liver function (bilirubin) and increased survival after liver transplantation (Figs. 7 and 8), indicating that SK2 also plays important role in I/R injury during LT and therefore is a novel therapeutic target for PNF.

The role of SKs in I/R injury is controversial. S1P administration and overexpression of SK1 by adenoviral gene transfer are reported to protect against myocardic I/R injury [49], [50]; whereas N,N,N-trimethylsphingosine, a SK inhibitor, also protects against myocardial I/R injury [60]. Deficiency of S1P_3_, a subtype of S1P receptor, decreases renal and pulmonary I/R injury [47], [48]. A recent study showed that S1P injection decreases hepatic and renal injury after hepatic I/R, possibly through S1P_1_ [61]. In contrast, FTY720 which after phosphorylation internalizes and down-regulates S1P receptors protects the liver from I/R injury [62], consistent with our results that inhibition of SK2 prevents liver injury after warm I/R [45] and after transplantation. Overall, the majority of the data suggests that SK inhibition and consequent reduction of S1P production and/or signaling protects against I/R injury. The discrepancies may be due to organ specific effects of SKs, relating to different subtypes of SKs or S1P receptors. Moreover, S1P metabolism in vivo could affect its biological effects. For example, serum phosphatases degrade injected S1P or dihydro-S1P to sphingosine and dihydrosphingosine, which is a SK inhibitor [63]. Moreover, injection of S1P could alter the expression and intracellular localization of S1P receptors. Therefore, the interpretation of the results of S1P administration should be taken with caution.

### Inhibition of SK2 Suppresses Innate Immunity-Mediated Inflammatory Processes after Liver Transplantation

Reperfusion injury involves both direct and indirect cytotoxic mechanisms. Numerous studies showed that inflammation occurs after I/R and LT, and multiple innate immune activation pathways are involved in the inflammatory responses [18]. In vivo, inflammatory responses after the initial I/R insult act to sustain and magnify the extent of I/R injury. Suppression of local immune activation consistently attenuates I/R injury [62], [64]–[67]. Previous studies showed that activation of SK results in a number of pro-inflammatory responses [36], [39]–[42], [68], [69]. SK is activated in inflammatory cells such as monocytes and macrophages [70], [71]. SK inhibition attenuates inflammatory diseases such as bacterial sepsis, ulcerative colitis, allergic asthma, anaphylaxis and arthritis in vivo [39], [42], [72], [73]. Our recent studies also showed that ABC294640 attenuates inflammatory responses after hepatic warm I/R [45].

A number of pathogen-associated and/or endogenous damage-associated molecular pattern (PAMP/DAMP) molecules are generated during cell stress and I/R. PAMP/DAMP bind to pattern recognition receptors (PRRs; e.g. TLRs and the receptor for advanced glycation end products), activating intracellular signaling pathways that initiate and/or sustain the inflammatory responses [18], [58], [74]–[76]. It appears that TLR4, but not TLR2 signaling, triggers liver inflammation [18]. Endotoxin (LPS) from intestinal flora, an exogenous PAMP, increases after LT due to gut hypoxia during surgery [21]. Endogenous DAMPs that activate liver TLRs during I/R are released from hypoxic or inflamed live cells and necrotic cells (e.g. high mobility group box-1; HMGB1) or derived from degraded extracellular matrix. HMGB1 protein is also secreted from activated KCs [18]. TLR4 in liver nonparenchymal cells is the main target for LPS and HMGB1 [26], [27], [77]. KCs play an essential role in liver I/R injury after LT [21]. TLR4 activation in KCs leads to ROS formation and NF-κB activation, which in turn increases toxic cytokines, proinflammatory enzymes such as cyclooxygenase-2 (COX-2), and adhesion molecules formation [26], [27], [77]. The absence of TLR4 in the donor organ reduces I/R injury after murine LT [78]. Sphingosine metabolism appears to regulate TLR4/NF-κB activation. S1P enhances protein kinase C-δ activation, a process that is required by TLR-dependent NF-κB activation [72]. In this study, we observed that ABC294640 suppresses TLR4 and ICAM-1 upregulation and NF-κB activation after transplantation (Fig. 2), consistent with the hypothesis that SK2 plays important roles in these key local inflammatory processes.

Formation of proinflammatory cytokines/chemokines stimulates subsequent expression of adhesion molecules and stimulates recruitment and activation of leukocytes. TNFα, a pivotal pro-inflammatory and cytotoxic cytokine, increases after LT, and the absence of TNFα receptors in the donor organ improves the outcome after LT in mice [79], [80]. S1P specifically binds to TRAF2, thus activating TNFα-induced downstream NF-κB signaling [81]. The non-specific SK inhibitor dimethylsphingosine blocks inflammatory cytokine-induced adhesion molecule expression in vitro [36]. Our previous studies also showed that SK2 inhibition decreases TNFα formation after warm I/R [45]. In the present study, we observed that formation of TNFα and IL-1β, another important proinflammatory cytokine, increased after LT, which was partially blocked by SK2 inhibition (Fig. 3). In addition, ABC294640 significantly decreased expression of CXCL-10 (Fig. 3), a chemokine that is involved in the attraction of monocytes/macrophages, T cells, NK cells and dendritic cells [55]. Therefore, SK2 affects inflammatory processes after transplantation also by regulation of formation of multiple cytokines/chemokines.

Resident macrophages and infiltrating inflammatory cells produce and release reactive oxygen and nitrogen species, hydrolytic enzymes (e.g. proteases, elastases) and toxic cytokines that further expand cell damage after I/R and LT [65], [78], [80], [82]–[84]. In this study, we observed infiltration of PMNs, monocytes/macrophages and expansion of resident macrophage population (KCs) in the liver after LT (Figs. 4 and 5), and all of these responses were blunted by ABC294640 treatment. These data are consistent with a notion that SK2 plays an important role in the inflammatory processes after LT.

### Inhibition of SK2 Suppresses T cell Trafficking after Liver Transplantation

Recent studies revealed that cross-talk between innate and adaptive immunities participate in the development and maintenance of I/R injury [18]. CD4+ T cells are crucial in promoting liver proinflammatory immune responses in I/R injury, even in syngeneic recipients in the sterile environment where exogenous T cell antigen was absent [31]. CD4+ T cell depletion or CD4-deficiency, but not CD8+ T cell depletion or targeted deficiency, are protective from I/R damage [31]. Inflammatory cytokines (e.g. TNFα) and chemokines and HIF-1α upregulation during hypoxia promote migration of CD4+ T lymphocytes to the liver after I/R [30], [31], [85]. CD4+ T cells can differentiate into multiple types of Th1, Th2, Th17 or Treg effectors. A number of studies showed that sphingosine kinases and S1P alter T cell function. For example, SEW2871, a S1P receptor agonist, stimulates lymphocyte trafficking in vitro [69]. S1PR1 facilitates T cell trafficking and retention in non-lymphoid tissues [86], [87]. In this study, we found that CD4+ T cells increased slightly after syngeneic LT in rats and this effect was blunted by ABC294640 **(** **Fig. 6** **)**. Therefore, SK2 also affects T cell trafficking and function after transplantation.

Taken together, inhibition of SK2 by ABC294640 protected against transplantation-induced inflammation and cross-talk between innate and adaptive immunities, major events precipitating and exacerbating graft injury. The increase in animal survival after transplant with ABC294640-treated grafts in combination with the extensive mechanistic analyses of the current study suggest that selective SK2 inhibition by ABC294640 represents a promising new strategy to attenuate liver graft injury in clinical settings. Although toxicity of ABC294640 in humans remains to be evaluated, acute and chronic toxicology studies in rats show that ABC294640 induces a transient decrease in the hematocrit (∼20%) after dosing at 100–250 mg/kg for 7 days; however, this normalizes after 28 days of treatment. Such changes are scored as grade 0 toxicity on the standard National Cancer Institute Scale for evaluating toxicity in clinical trials. No other changes in hematological parameters, clinical chemistry, coagulation parameters, or gross and microscopic tissue pathology result from treatment with ABC294640 [53]. For application in transplantation, ABC294640 is dissolved in the cold storage solution. Therefore, its toxicity to graft recipients would likely be minimal.
